# Treatment strategies for unruptured intracranial aneurysms in the Chinese population: China Treatment Trial for Unruptured Intracranial Aneurysm (ChTUIA)

**DOI:** 10.1186/s41016-025-00394-7

**Published:** 2025-04-03

**Authors:** Kaige Zheng, Zheng Wen, Kaiwen Wang, Shaohua Mo, Jun Wu, Xiaolin Chen, Bing Zhao, Qingyuan Liu, Shuo Wang

**Affiliations:** 1 Department of Neurosurgery, Beijing Tiantan Hospital, Capital Medical University, Beijing, China; 2 China National Clinical Research Center for Neurological Diseases, Beijing, China; 3 Department of Neurosurgery, Beijing Friendship Hospital, Capital Medical University, Beijing, China; 4 Department of Neurosurgery, Renji Hospital Affiliated to Shanghai Jiao Tong University School of Medicine, Shanghai, China

**Keywords:** Unruptured intracranial aneurysm, Treatment, Endovascular treatment, Surgical clipping, Complication

## Abstract

**Background:**

Intracranial aneurysm is a leading cause of subarachnoid hemorrhage and affects approximately 7% of the Chinese population, posing a significant public health concern. Due to the lack of a national cohort of unruptured intracranial aneurysms (UIAs) in China, optimal surgical management for UIAs remain insufficiently explored.

**Methods:**

The China Treatment Trial for Unruptured Intracranial Aneurysm (ChTUIA) is a national, prospective, observational, multi-center registry study designed to identify optimal surgical management for UIAs in the Chinese population. Eligible patients were recruited from 83 regional neurological centers in China between December 2021 and December 2022. All patients will be followed up routinely for at least two years.

**Results:**

A total of 25,438 patients with UIAs have been enrolled in the study, of whom 9794 (38.5%) were male, with a median age of 59 years (interquartile range: 52–67 years). The mean follow-up period was 4.28 years (interquartile range: 2.86–6.37 years). Among the patients, 6,712 (26.4%) patients underwent microsurgical clipping, and 18,726 (73.6%) patients underwent endovascular treatment, including 3,017 (16.1%) receiving coil alone or balloon-assisted coiling, 11,431 (61.1%) underwent stent-assisted coiling, and 4,278 (22.8%) treated with flow diverters. Comprehensive data collection encompassed 874,890 demographic and clinical records, 42,109 radiological records, 13,528 hemodynamic records, and 12,727 biological records, with a lost-to-follow-up rate of 5.4% and a data-missing rate of 8.3%.

**Conclusions:**

The ChTUIA study represents the first national prospective investigation into surgical management protocols and treatment trends toward UIAs in the Chinese population. This study will provide critical evidence to guide the clinical management of UIA patients.

**Trial registration:**

NCT05844163 (https://clinicaltrials.gov/study/NCT05844163 ).

## Background

Unruptured intracranial aneurysms (UIAs) are pathological dilations in cerebral arteries and represent a leading cause of non-traumatic subarachnoid hemorrhage [1, 2]. Previous studies have reported that UIAs are present in 7% of Chinese individuals aged 35–75 years [3]. With an aging population and advances in radiological technology, the UIAs has been increasingly detected in the Chinese population. Aggressive treatment of UIAs is still controversial because of a relatively low risk of rupture and a high risk of interventional treatment. However, UIAs may rupture and lead to subarachnoid hemorrhage (SAH) in a subset of cases, with the mortality rate after SAH occurrence being around 50% [4].

**Fig. 1:**
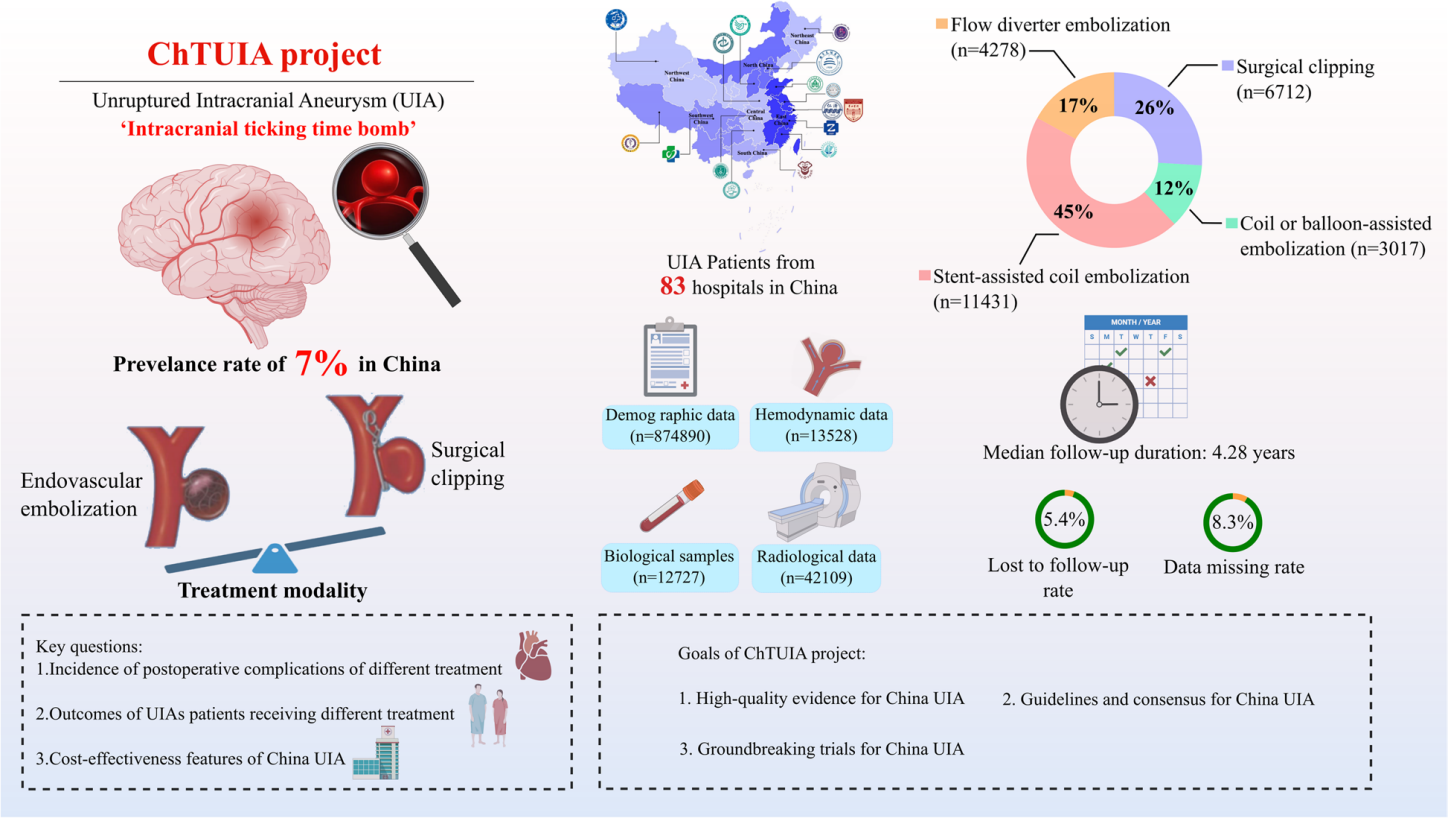
Overview of the ChTUIA study. Abbreviations: UIA, unruptured intracranial aneurysm

Current interventional strategies to prevent UIA rupture include microsurgical clipping and endovascular treatment [5, 6]. Preventive treatments for UIAs with high risk of rupture are essential for reducing the morbidity and mortality caused by subarachnoid hemorrhage. However, the optimal surgical management strategies for UIAs remain underexplored due to the absence of national prospective cohort studies on UIAs in the Chinese population. The China Treatment Trial for Unruptured Intracranial Aneurysm (ChTUIA) is a national, prospective, observational, and multi-center registry study. This study will investigate the optimal surgical management strategies for UIAs and provide high-quality evidence of clinical practices for UIAs in China (Fig. 1).

## Methods

### Study design and patients

**Fig. 2:**
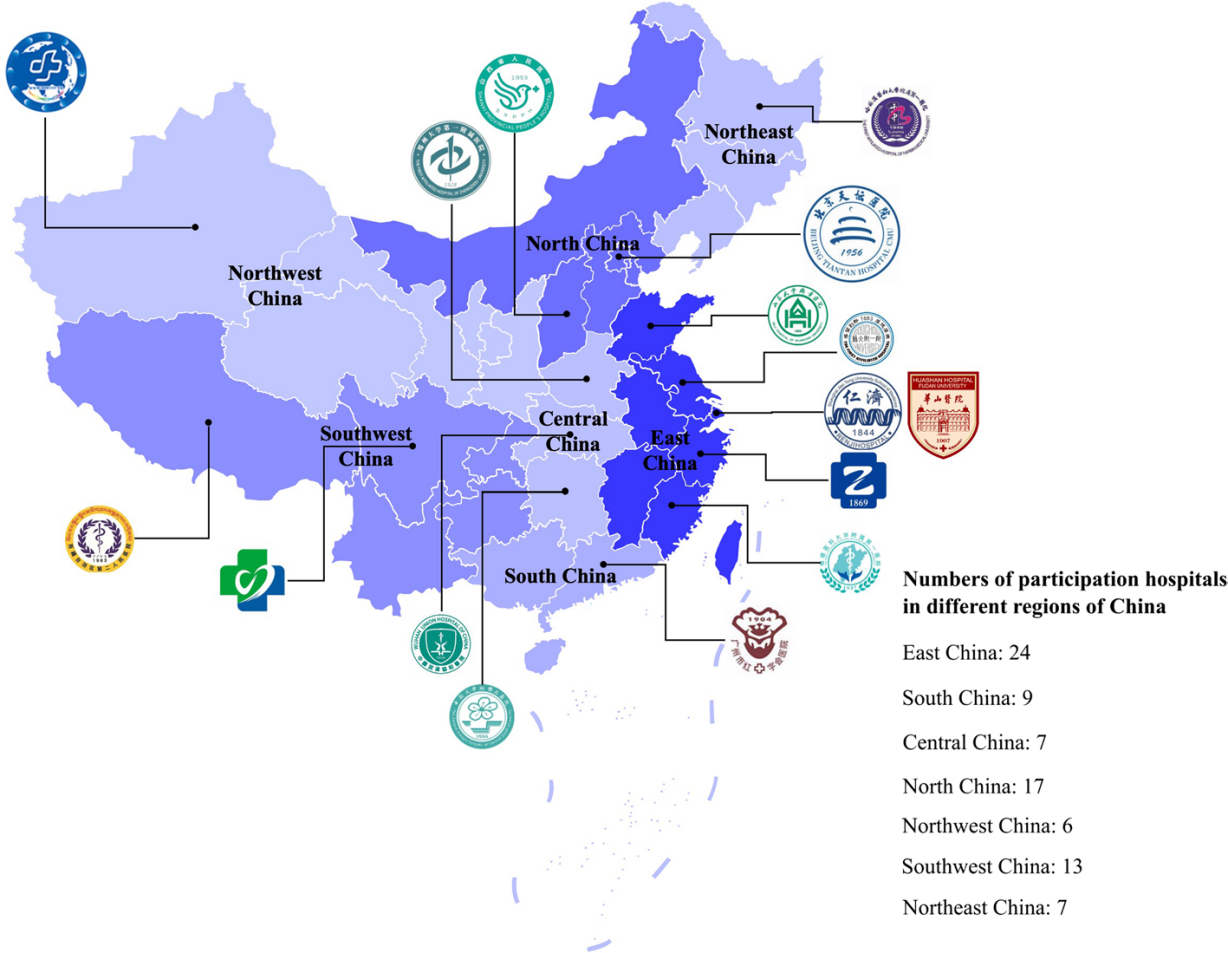
The geographic locations of participating hospitals in the ChTUIA

**Fig. 3:**
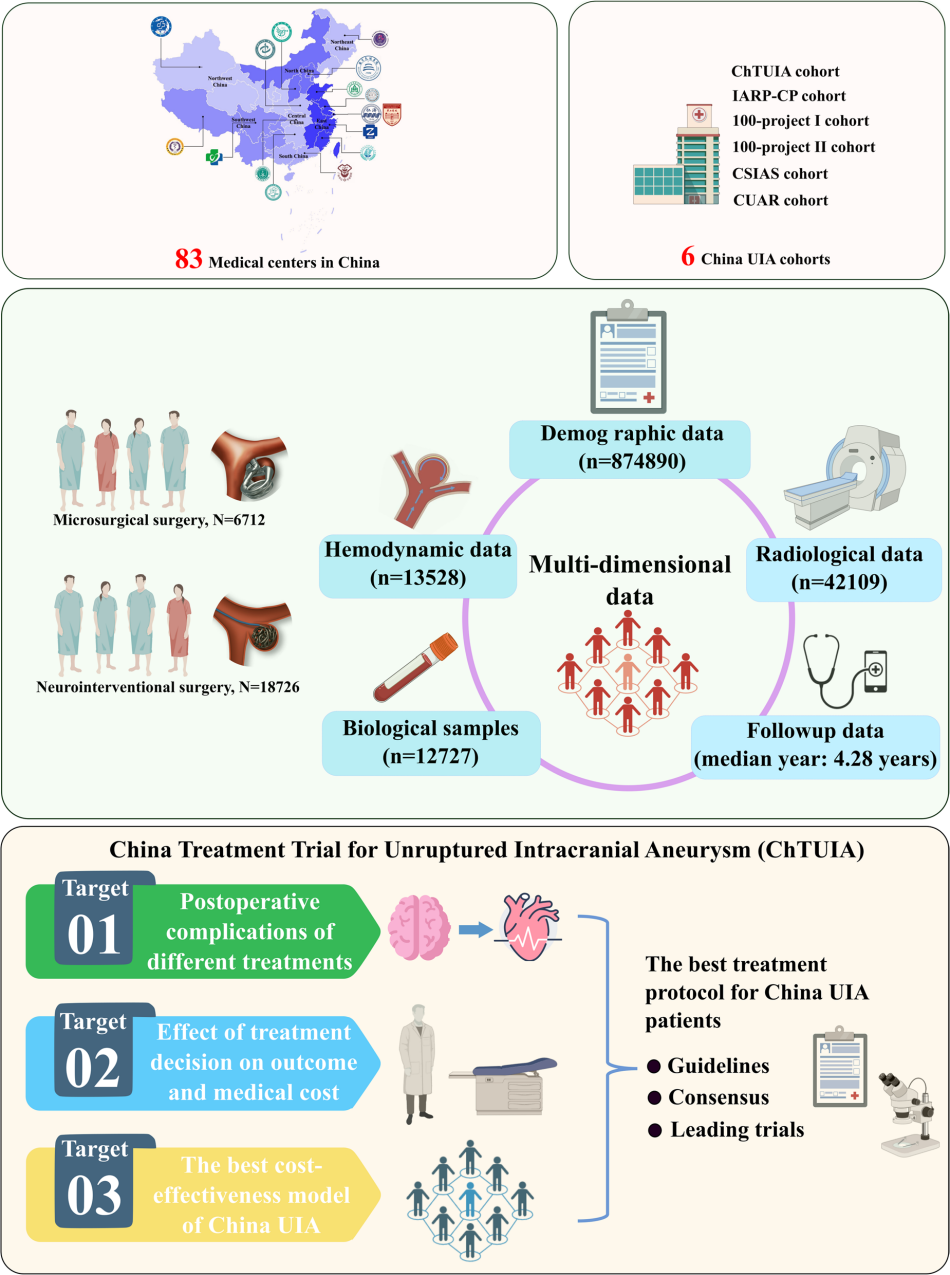
The flowchart of ChTUIA study. Abbreviations: UIA, unruptured intracranial aneurysm

The ChTUIA study is a national, prospective, observational, multi-center registry that focusing on patients with UIA receiving any surgical treatment or endovascular treatment at 83 regional neurological medical centers across China, conducting from December 2021 to December 2024 (Fig. 2). Eligible patients were continuously recruited between December 2021 and December 2022. The flowchart for ChTUIA is presented in Fig. 3. Inclusion criteria were: (1) age more than 18 years, (2) at least one unruptured intracranial aneurysm treated by surgical, endovascular treatment or combined treatment. (3) patients’ informed consent was obtained. Exclusion criteria were: (1) ruptured intracranial aneurysm, subarachnoid hemorrhage, or intracerebral hemorrhage; (2) intracranial aneurysms related to trauma, infection, or atrial myxoma; (3). combined with cerebrovascular malformations; (4). combined with brain tumors or malignant tumors in other parts; (5). combined with systemic connective tissue disease and systemic rheumatic disease; (6). Due to other diseases, or poor general condition, the expected survival time is not more than 12 months; (7). During pregnancy and perinatal period.

### Management protocols

Management decision for UIAs have been made according to the physician evaluation and patient wishes. Treatment modalities were performed routinely in each center. All enrolled UIA patients received one of the following treatments: (1) microsurgical clipping; (2) coil embolization alone or balloon-assisted embolization; (3) stent-assisted coil embolization; or (4) flow diverter placement. Based on these interventions, patients were categorized into two main groups: the microsurgical clipping group and the endovascular coiling group. For subgroup analysis, the endovascular treatment was further divided into three specific categories based on the treatment modalities.

### Data collection

As shown in Table 1, the study captures five categories of features from UIA patients, including demographic and clinical, radiological, hemodynamic, biological, and follow-up data. Demographics (e.g., sex, age, ethnicity, family history, permanent residence, place of origin), lifestyle factors (e.g., alcohol and tobacco use, occupation, education level), comorbidities (e.g., hypertension, diabetes mellitus, dyslipidemia, cardiovascular and cerebrovascular disease, and previous medication usage), family history (e.g., Marfan syndrome, polycystic kidney disease, Ehlers-Danlos Syndrome), radiological findings (e.g., multiple aneurysms, aneurysm location, size, shape), physical and neurological assessments (e.g., preoperative/postoperative mRS), laboratory findings, treatment details (e.g., type of surgery, surgery duration, intraoperative hemorrhagic and ischemic events, aneurysm occlusion classification by Raymond classification, blood transfusion requirements, postoperative medication, post-treatment complications; For patients with multiple aneurysms, staged treatment information for multiple aneurysms were also recorded), and follow-up information (e.g., neurological function, incidence of MACCE events, mortality, aneurysm recurrence).

**Table 1:** Data collection at different stages of UIA treatment

**Characteristics**	**Screening**	**Pre-treatment**	**Treatment period**	**Before discharge**	**Follow-up**
Diagnosis	×				
Written informed consent	×				
Demographic data	×				
Life-style information	×				
Medical history	×				
Physical examination		×	×		
Neurological function		×	×		×
Laboratory examination		×	×		
Imaging examination		×	×	×	×
Treatment strategy			×		
Post-treatment complications				×	×
Cadiovascular and cerebrovascular event			×	×	×
Hemorragic events			×	×	×
UIA-related cost				×	×

*Abbreviations:* *UIA* Unruptured intracranial aneurysm

Data collection is conducted by centralized, trained personnel adhering to a standardized protocol established by the Organizing Committee of the ChTUIA study. Once the data has been gathered at each center, it is uploaded to a centralized Data Committee for further processing. An electronic data capture (EDC) system was developed facilitating structured data entry via a web-based platform. An independent data committee, comprising neurosurgeons, radiologists, and statisticians, conducts regular monitoring through the EDC to identify and address any data inconsistencies or errors. All data undergo double-checking by clinicians and statisticians and are anonymized before or during analysis.

### Radiological data collection

**Fig. 4:**
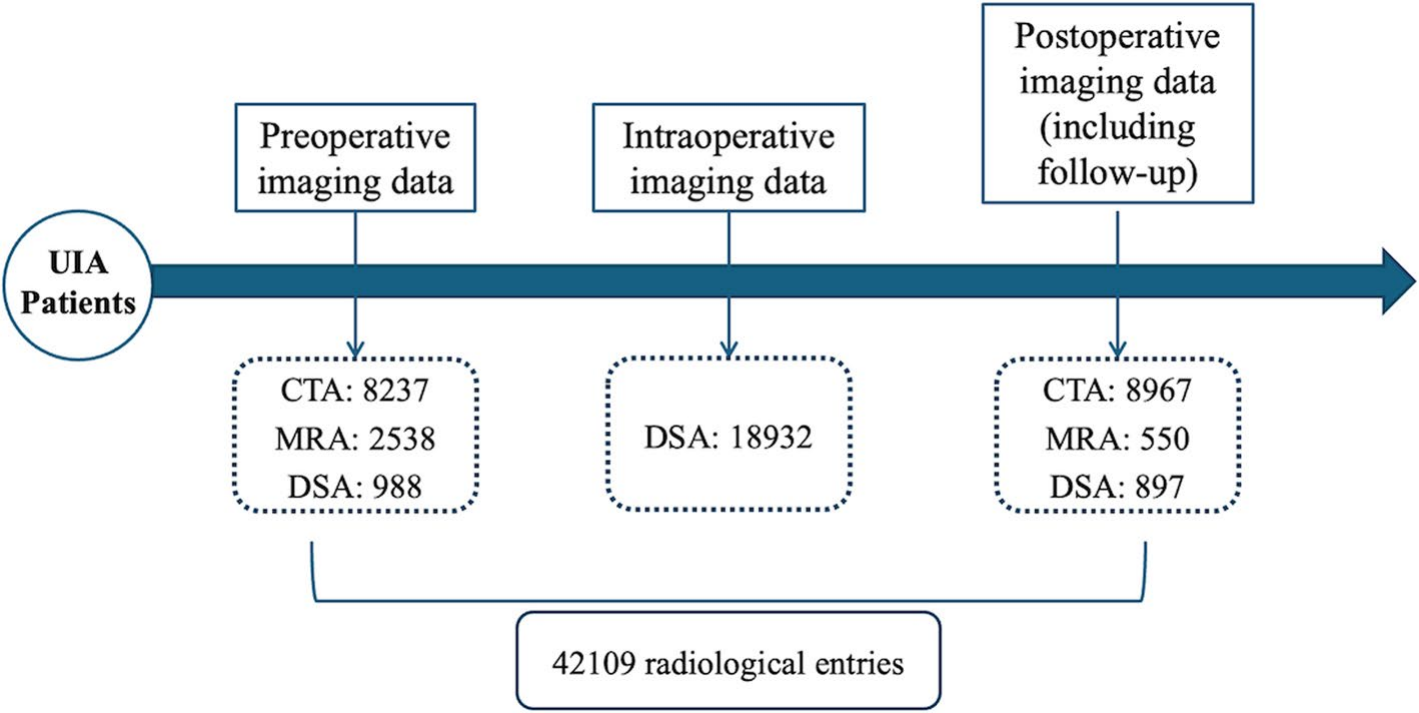
Radiological data collection of ChTUIA study. Abbreviations: UIA, unruptured intracranial aneurysm; CTA, Computed Tomography Angiography; MRA, Magnetic Resonance Angiography; DSA, Digital Subtraction Angiography

Radiological data from admission to follow-up were collected by the CRO in the original DICOM format. These files are then subjected to quality control and de-identification before undergoing centralized review. The imaging review results are audited and confirmed by the Imaging Review Committee and subsequently entered the imaging database system (Fig. 4).

### Biological sample collection and storage

Blood samples were collected within 48 h before surgery. To ensure consistency, all patients were required to fast for 8 h before blood sampling. After excluding blood samples with hemolysis, the reminder was centrifuged at 3000 rpm for 10 min within 3 h of collection. With the tissues carefully washed with low-temperature phosphate-buffered saline to remove blood cells within the vessels, both tissues and serums were finally obtained and stored in liquid nitrogen.

### Primary and secondary outcomes

The primary outcome was the neurological functional outcome of UIA patients at two years of follow-up after treatment. Neurological outcome was evaluated using the modified Rankin Scale (mRS), where a mRS score of 2–6 was classified as a poor outcome, and a score below 2 as a good outcome.

Secondary outcomes included: (1) patients' neurological function outcomes at one year after treatment post-surgery; (2) incidence of major cardiovascular and cerebrovascular events within one year post-surgery in UIA patients with coronary heart disease; (3) incidence of cardiovascular or cerebrovascular events within two year after treatment; (4) all costs associated with UIA treatment within two years after treatment; (5) incidence of aneurysm-related bleeding events within two years post-surgery; (6) UIA-related mortality within two years; and (7) all-cause mortality rate within two years after undergoing different surgical treatments.

### Follow-up assessment

Patients are followed up every three months during the first year and every six months thereafter, conducted by trained clinical research coordinators independent any treatment through outpatient visits or phone calls. To minimize the risk of follow-up loss, at least three contact numbers are recorded for each patient. During follow-up, coordinators document neurological function, the incidence of any cardiovascular and cerebrovascular events, mortality, aneurysm recurrence, and associated healthcare costs. If a patient is suspected of reaching a primary or secondary outcome, their medical records, radiological data, and laboratory results are collected for further assessment. An independent monitoring committee verifies the occurrence of primary and secondary outcomes to ensure data accuracy.

### Statistical analysis

Statistical analysis was conducted using SAS software (version 9.4, SAS Institute, USA) and SPSS (version 25, IBM Institute, USA). Categorical variables are presented as percentages, while continuous variables are reported as means with standard deviations (SDs) or medians with interquartile ranges (IQRs). To compare continuous variables, either the *t* test or Mann–Whitney test was applied, depending on data distribution. For categorical variables, the χ^2^ test or Fisher’s exact test was used. Univariate and multivariate logistic regression analyses were performed to assess factors associated with long-term neurological outcomes, with ORs and their 95% CIs evaluated. A *p* value of < 0.05 was considered statistically significant. This study also included a comparison of baseline demographic, clinical, and imaging characteristics among UIA patients receiving different treatments modalities.

### Ethic and dissemination

This study was approved by the Institutional Review Board of Tiantan Hospital, with strict adherence to the Declaration of Helsinki throughout the research. Patient information has been kept confidential during the study and the publication process.

## Results

A total of 37,806 patients with UIAs provided consent and were included in the ChTUIA study from 83 regional neurosurgery centers between 2021 and 2024. Following rigorous screening and elaborative follow-up, 25,438 patients were ultimately enrolled. These data included 874,890 demographic and clinical entries, 42,109 radiological entries, 13,528 hemodynamic entries, and 12,727 biological entries, with a 5.4% lost-to-follow-up rate and an 8.3% data-missing rate (Table 2).

**Table 2:** Data collection at different centers of ChTUIA

	UIA patients n (%)	Imaging data n (%)	Biological sample n (%)
	13,467 (52.9%)	24,998 (59.4%)	8235 (64.7%)
	4532 (17.8%)	7459 (17.7%)	1537 (12.1%)
	2237 (8.8%)	2976 (7.1%)	1052 (8.3%)
	1784 (7.0%)	2653 (6.3%)	821 (6.5%)
Other Centers	3418 (13.4%)	4023 (9.6%)	1082 (8.5%)

*Abbreviations*: *UIA *Unruptured intracranial aneurysm

Overall, 9795 (38.5%) patients were male, with a median age of 59 years (IQR, 52–67 years). Surgical clipping was performed on 6,712 (26.4%) patients, while 18,726 (73.6%) underwent endovascular treatment. Among those receiving endovascular treatment, 3,017 (16.1%) received coil or balloon-assisted embolization, 11,431 (61.1%) received stent-assisted coil embolization, and 4,278 (22.8%) were treated with a flow diverter.

## Discussion

This study is a national, prospective, observational, multicenter registry study focusing on surgical treatment of UIAs and two years of long-term outcome across in China. This study adhered to stringent quality control and tracking measures, achieving low rates of lost-to-follow-up and data-missing. This study enables it to comprehensively document outcomes and benefits for UIA patients with different treatment modalities, providing valuable evidence for UIA management in clinical practice.

Many factors, including patient election, advanced age, medical history, and aneurysm characteristics and physician’s experience may influence clinical treatment strategies for UIAs. The Chinese population have a high prevalence of risk factors associated with ischemic cardio- and cerebrovascular conditions, including hypertension, diabetes, and arteriosclerosis [7–9]. This predisposition significantly increases the risk of ischemic events after aneurysm treatment. Given the complex nature of these complications, there is an urgent need for a comprehensive framework to assess and manage postoperative complications in clinical settings. This study will investigate the treatment related to complications, including ischemic stroke, headache, and neurological deficits.

With advancements in aneurysm treatment techniques and technologies, surgical treatments of UIAs are rapidly evolving. The ISUIA study and several meta-analyses indicate that while surgical clipping offers a high aneurysm occlusion rate and a lower recurrence rate, it is associated with high risk of treatment related to complications. Conversely, endovascular treatment is less invasive but has a higher recurrence rate [5, 6]. As a result, the optimal treatment strategy for UIAs remains a topic of ongoing debate. By assessing outcomes and the presence of postoperative complications in patients receiving various treatments, the ChTUIA study will identify which UIA patients benefit from surgical or endovascular treatment across different patient groups to provide more informed decision-making in UIAs management.

The decision to intervene in UIAs is based on careful evaluation of the risks and benefits associated with treatment. Key considerations include the patient’s overall health, the aneurysm’s size and location, estimated rupture risk, and the potential for treatment-related complications. Symptomatic UIAs, particularly those presenting with sentinel headaches or cranial nerve deficits, often indicate a higher risk of rupture and may warrant surgical intervention. Conversely, small, asymptomatic aneurysms with low rupture risk may be managed conservatively through regular imaging surveillance, which has been shown to be a cost-effective strategy in certain populations. By integrating multimodal data, including detailed clinical information, advanced radiological imaging, and biological biomarkers. ChTUIA study seeks to identify predictive factors that can optimize decision-making, balancing the risks of intervention against the natural history of the aneurysm, ultimately improving patient outcomes.

Although substantial studies have evaluated the cost-effectiveness of surgical clipping and endovascular treatment in UIAs management using prospective cohorts, these studies mainly focused on mortality and morbidity due to aneurysm rupture [10–12]. Long-term outcomes following treatment and the postoperative complications data are limited. The ChTUIA study will assess the cost-effectiveness of UIA treatment and identify those who would benefit most from different interventions, and treatment establish an evidence-based cost-effectiveness model tailored to the Chinese population.

## Conclusions

Due to the absence of a national cohort of UIA patients undergoing optimal management protocols for UIAs remain unclear in China. the ChTUIA is a nationwide, prospective, observational, multicenter study to generate high-quality evidence for UIA treatment in China; and will guide treatment-decision making in the Chinese population.

## Data Availability

The data supporting the findings of this study are available from the corresponding author s upon reasonable request.
