# Correction: Treatment strategies for unruptured intracranial aneurysms in the Chinese population: China Treatment Trial for Unruptured Intracranial Aneurysm (ChTUIA)

**DOI:** 10.1186/s41016-025-00404-8

**Published:** 2025-08-20

**Authors:** Kaige Zheng, Zheng Wen, Kaiwen Wang, Shaohua Mo, Jun Wu, Xiaolin Chen, Bing Zhao, Qingyuan Liu, Shuo Wang

**Affiliations:** 1 Department of Neurosurgery, Beijing Tiantan Hospital, Capital Medical University, Beijing, China; 2 China National Clinical Research Center for Neurological Diseases, Beijing, China; 3 Department of Neurosurgery, Beijing Friendship Hospital, Capital Medical University, Beijing, China; 4 Department of Neurosurgery, Renji Hospital Affiliated to Shanghai Jiao Tong University School of Medicine, Shanghai, China

**Correction: Chinese Neurosurgical Journal 11, 8 (2025)**

**https://doi.org/10.1186/s41016-025-00394-7**

Following publication of the original article [1], the authors reported that the first sentence under the Study design and patients section needed to be updated. This minor revision does not change the experimental design, results, or conclusions of the study. Its sole purpose is to improve the clarity of the description and prevent any potential misinterpretation by the readers.

The Original sentence is:

The ChTUIA study is a national, prospective, observational, multi-center registry that focusing on patients with UIA receiving any surgical treatment or endovascular treatment at 83 regional neurological medical centers across China, from December 2021 to December 2024 (Fig. 2).

The revised sentences are:

The ChTUIA study is a national, prospective, observational, multi-center registry that focusing on patients with UIA receiving any surgical treatment or endovascular treatment at 83 regional neurological medical centers across China, conducting from December 2021 to December 2024 (Fig. 2). Eligible patients were continuously recruited between December 2021 and December 2022.

The original article [1] has been corrected.

## References

[1] Zheng K, Wen Z, Wang K, et al. Treatment strategies for unruptured intracranial aneurysms in the Chinese population: China treatment trial for unruptured intracranial aneurysm (ChTUIA). Chin Neurosurg Jl. 2025;11:8. 10.1186/s41016-025-00394-7. 10.1186/s41016-025-00394-7 PMC11969722 40181400

